# A multi-objective constraint-based approach for modeling genome-scale microbial ecosystems

**DOI:** 10.1371/journal.pone.0171744

**Published:** 2017-02-10

**Authors:** Marko Budinich, Jérémie Bourdon, Abdelhalim Larhlimi, Damien Eveillard

**Affiliations:** Computational Biology group, LINA UMR 6241 CNRS, EMN, Université de Nantes, Nantes, France; Tel Aviv University, ISRAEL

## Abstract

Interplay within microbial communities impacts ecosystems on several scales, and elucidation of the consequent effects is a difficult task in ecology. In particular, the integration of genome-scale data within quantitative models of microbial ecosystems remains elusive. This study advocates the use of constraint-based modeling to build predictive models from recent high-resolution -omics datasets. Following recent studies that have demonstrated the accuracy of constraint-based models (CBMs) for simulating single-strain metabolic networks, we sought to study microbial ecosystems as a combination of single-strain metabolic networks that exchange nutrients. This study presents two multi-objective extensions of CBMs for modeling communities: multi-objective flux balance analysis (MO-FBA) and multi-objective flux variability analysis (MO-FVA). Both methods were applied to a hot spring mat model ecosystem. As a result, multiple trade-offs between nutrients and growth rates, as well as thermodynamically favorable relative abundances at community level, were emphasized. We expect this approach to be used for integrating genomic information in microbial ecosystems. Following models will provide insights about behaviors (including diversity) that take place at the ecosystem scale.

## Introduction

Microbial organisms comprise approximately 50% of the Earth’s biomass [1, 2] and their interplay drives most biogeochemical cycles [3, 4]. The study of microbial interactions, which occur at the molecular scale, remains crucial to the elucidation of larger-scale processes [5]. Several models have attempted to simulate the quantitative impact of molecular-scale processes at an ecosystem level. Among others, trait-based approaches have gained attention as a precise way to understand and predict the quantitative behaviors of microbial communities [6, 7]. However, such models remain difficult to apply to most communities without the additional expertise required for deciphering particular traits and performing extensive experiments to design accurate parameters [8]; such expertise is often unavailable for the study of natural communities.

In the last decade, great advances have been made in the development of high-throughput techniques that enable the study of the metagenomics, meta-transcriptomics, and meta-metabolomics of natural communities. Such techniques provide ‘omics-scale information for organisms, from which it is possible to identify specific molecules (*e.g.,* DNA, mRNA, metabolites) present in a particular microbial ecosystem. Such studies of microbial ecosystems have facilitated drastic changes in approaches utilized for characterizing microbial communities [9, 10], thus leading to the emergence of the field of microbial systems ecology. Further, advances in bioinformatics and computational techniques have enabled the development of next-generation sequencing technologies for the qualitative analysis of microbial environments by emphasizing *who is there and who is not* [11] and allowing the study of the co-existence of microbial strains under different environmental conditions (see [12] for illustration). However, among the most significant challenges in modeling microbial communities remains the ability to quantitatively predict microbial community composition and functions under specific environmental conditions.

We propose to overcome this challenge by using recent systems biology approaches for the prediction of quantitative behaviors of single organisms based on genome-scale data [13, 14]. This study presents a natural extension of such approaches via their application to the modeling of microbial ecosystems and the elucidation of their quantitative features [15, 16].

Genome-scale descriptions, in this context, are provided by metabolic networks. A metabolic network summarizes the set of biochemical reactions encoded by the genome of a given organism. Two reactions are linked within a metabolic network if the substrate of one reaction is the product of the other. Such genome-scale descriptions of organisms are currently applied in systems biology for the purpose of investigating physiology [17]. In particular, for an increasing number of species, current bioinformatics protocols build genome-scale metabolic networks from genome-scale transcriptomic or metabolomic data [18].

Quantitative analyses utilize such metabolic networks as inputs for constraint-based models (CBMs) in order to infer physiological features based on a genome-scale description [17]. As a central assumption, constraint-based modeling considers the constraints defined by the set of reactions as linked within a metabolic network at steady state, and assume the corresponding model to behave optimally to achieve a given objective [13, 14]. The use of constraint-based modeling for microbial ecosystems, which involves the generation of a framework to perform data integration as well as mathematical descriptions useful for numerical simulations, seems promising [16, 19].

Several attempts have been made to model the metabolic network of microbial communities. Rodrígez *et al*. [20] proposed to use a “supra-organism” assumption, which considers reactions of all members of the community as a single entity. While such an approximation was used in recent studies (see Biggs *et al.* [21] and Perez-Garcia *et al*. [22] for a review), Kiltgord and Segré [23] previously showed that fluxes from a compartmentalized network and its de-compartmentalized counterpart (*i.e.*, supra-organism approach) are significantly different in their predicted FBA and FVA values. Furthermore, they show that fluxes using both assumptions are often not correlated. Such a distinction between both modeling results, along with the indisputable presence of compartments within ecosystems, clearly advocates for the use of compartments in the modeling. Considering so, several modelings have been proposed. However, while they all assume to consider distinct compartment for each microbial strain involved, they differ in their use of choosing the objective function. Stolyar et al. [24] first proposed a compartmentalized flux balance approach for modeling a mutualistic co-culture that requires an “ecosystem function”. Such a function is usually a weighted sum of each compartment objective. Nevertheless, the relative weight of each strain objective function remains herein at the discretion of an empirical expertise that is mostly out of reach for complex or uncharacterized microbial ecosystems.

To overcome such a weakness, more elaborated modeling approaches have been proposed. Zomorrodi and collaborators [25, 26] modeled each organism in a microbial community as a single CBM with its own objective function, nested within a global ecosystem model, thereby enabling the maximization of an ecosystem objective function. This approach still require to design an ecosystem objective function but proposes a multi-level optimization that considers both microbial strain and ecosystem objectives. Meanwhile, Khandelwal and collaborators [27] (followed by [28]) advocates for the use of the “balanced growth” concept, according to which all microorganisms grow at the same rate. Accordingly, this approach considers several compartment with no ecosystem objective function per se but rather introduces community fractions into the formulation, adding new degrees of freedom to the general optimization problem. Worth noticing, such a modeling assumption is justified for microbial communities for which biomass production is monitored and constrained in chemostat, but not necessary for open systems as observed in nature.

In this study, we propose a complementary model, to investigate the general case of microbial ecosystems. Based on Pareto optimality [29], we aim at describing all the feasible solutions considering metabolic constraints from each strain with no design of ecosystem function. Consistent with previous works, the present study considers the community as a compartmentalized system in which each organism (*i.e.*, a compartment) has (i) its own objective to optimize and (ii) shares metabolites through the environment. Contrary to above methods, our approach is based on multi-objective optimization, which allows us to consider the objective function of each organism simultaneously.

Specifically, following previous works, we implemented a multi-objective flux balance analysis method [30], henceforth known as MO-FBA, for microbial communities, which is based on an exact resolution algorithm. Additionally, we introduced a complementary multi-objective flux variability analysis (MO-FVA) method. These analyses emphasize putative metabolic behaviors that are optimal at the community level, while considering metabolic constraints for each strain. Finally, we performed complementary thermodynamics analysis [31], which enabled us to pinpoint (i) favored ecosystem responses to environmental parameters and (ii) the corresponding diversity.

For the sake of MO-FBA and MO-FVA illustration, this study models a microbial ecosystem comprising three distinct phenotypes: a primary producer, *Synecococcus spp.* (SYN), filamentous anoxygenic producers (FAP), namely *Chloroflexus spp.* and *Roseiflexus spp.*; and sulfate-reducing bacteria (SRB, composed by *Thermodesulfovibrio spp.*-like activity, [32]), as described in [33]. Results emphasize trade-offs between distinct bacterial growth rates based not only on environmental conditions and genome-scale descriptions of each strain, but also thermodynamical quantitative predictions that are consistent with experimental knowledge.

## Material and methods

### Metabolic networks as constraint-based models

**Fig 1:**
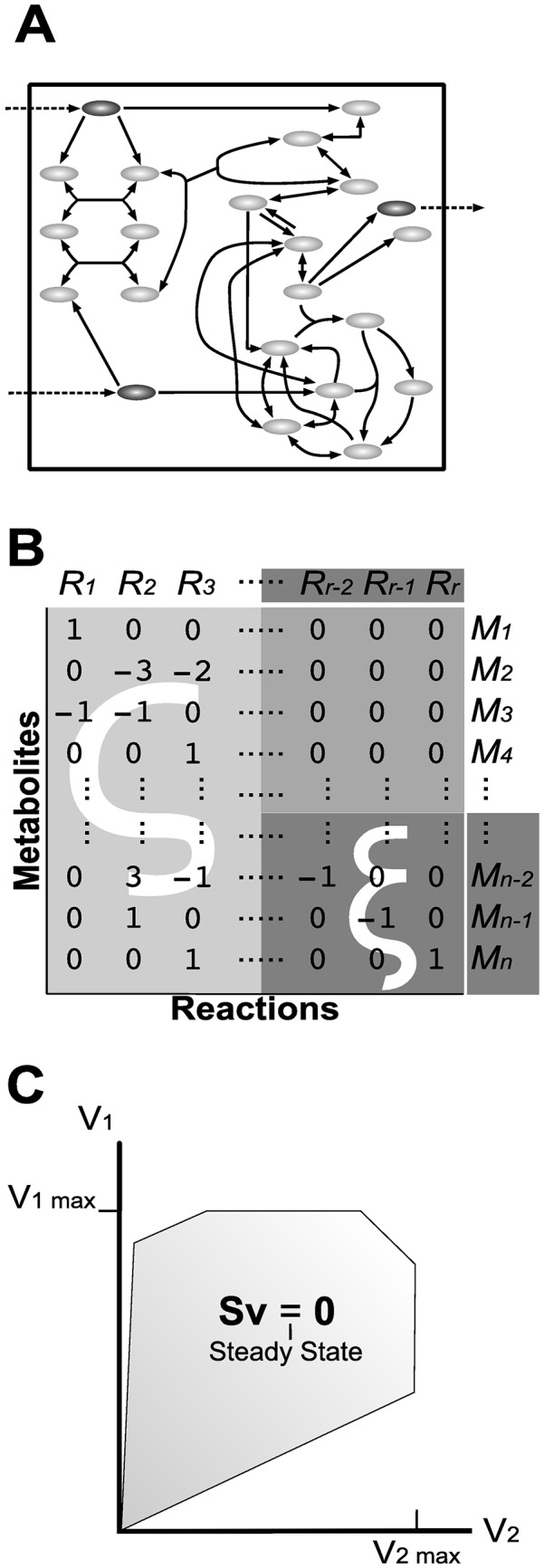
Construction of a Constraint Based Model (CBM). (A) **Metabolic Network** is represented as a chart of metabolites (ellipses) trough chemical reactions (arrows); borders represent the system boundary. (B) depicts the **Stoichiometric Matrix**, in which reactions are presented as columns and metabolites as rows. Each coefficient **S**_**ij**_ of the matrix corresponds to the stoichiometric coefficient of metabolite *M*_*i*_ in reaction *R*_*j*_, with reactants as negative and products positive. Exchange reactions and exchange metabolites are placed in the right and inferior section of the matrix, respectively. Therefore, submatrix *ς* is in the left and highlighted in light gray while submatrix *ξ* is highlighted in dark grey (see text). Normal gray depicts a matrix with only zeros. (C) **Flux space**, also known as “solution space”, is defined by the set of restrictions of the CBM (mass balance in steady state, bounded reaction rates, etc.) and contains all possible values of **v**.

The genomic data for a particular microorganism describes a set of genes, allowing the identification of enzymes and related reactions. Reactions produce metabolites that are used as substrates in subsequent reactions; such interplay constitutes a *“metabolic network”* whose size may vary from few tens to several hundreds of reactions [14]. Metabolic networks are modeled (Fig 1A) in order to study the physiology of the relevant microorganism. In particular, metabolic models are used to infer reaction rates, also known as fluxes, without using kinetic parameters. For this purpose, a metabolic model is formally described by its stoichiometric matrix **S** (Fig 1B), where the rows correspond to the metabolites and the columns correspond to the reactions considered in the metabolic network. At steady-state conditions, the rate of formation of internal metabolites is equal to the rate of their consumption. This is expressed by the flux balance equation **Sv** = 0, where **v** = (*v*_1_, …, *v*_*r*_) stands for the flux vector, *i.e.*, *v*_*j*_ is the flux of reaction *R*_*j*_ for all *j* = 1, …, *r*.

Under steady-state conditions, the continuous supply of metabolites from the media is facilitated by exchange reactions at a constant rate (dark gray eclipses and dashed lines in Fig 1A and highlighted dark gray block in Fig 1B). This matter exchange with the media allows the metabolic network to be in a non-equilibrium steady state (NESS). If metabolite exchange were not possible, then for each reaction the only possible state would be the chemical equilibrium, with all net fluxes equal to zero [31]. In the following, *ς* and *ξ* represent, respectively, internal reaction and exchange reaction submatrices (light gray and dark gray blocks in Fig 1B, respectively). Occasionally, exchange rates may be experimentally measured and incorporated into the model as equations of the form *v*_*i*_ = *b* for reaction *i*. In addition, maximal and minimal flux values may be expressed as *lower* and *upper* bounds constraints, by equations of the form *l*_*i*_ ≤ *v*_*i*_ ≤ *u*_*i*_, resulting in a model described as a set of constraints. Such models are termed CBMs. CBMs usually comprise more reactions than metabolites; therefore, these models are undetermined in that when a solution **v** exists, it is not unique. All feasible solutions define a *“flux space”* (Fig 1C) that may be further analyzed through several state-of-the-art approaches. For a detailed review of these methods, the reader may wish to refer to [13] and [14].

#### Flux balance analysis

Flux balance analysis (FBA) is one of the most widely used approaches for the identification of points of interest in the flux space [14]. Using this method, an objective function (for example, biomass production) is stated and its maximal value within the flux space is determined. In addition to the flux balance constraints, FBA utilizes flux capacity constraints that limit the fluxes of reactions. An optimal flux vector may be obtained by solving the following linear program (LP):

$$\begin{array}{ccccc} \underset{v\;\in \;R^{n}}{\text{maximize}} & & z=c^{⊺}v & & \\ \text{subject}\;\text{to} & & & & \\ & & \text{Sv}=0 & & \\ & & l_{i}\leq v_{i}\leq u_{i} & & i=1,\ldots ,n, \end{array}$$

where **c**^⊺^**v** is a linear combination of fluxes that represents the objective function (*i.e.*, biomass production or growth rate). From linear programming theory, it is known that the optimal value z* of objective function is unique; however, multiple flux distributions (*i.e.*, values of **v**) that achieve the same optimal value z* may exist.

#### Flux variability analysis

The set of all optimal flux distributions, *i.e.,* those with an optimal objective value of z*, may be investigated by using Flux Variability Analysis (FVA) to determine the flux range of each reaction in the metabolic network [14]. Formally, FVA solves the two following LPs for each reaction *R*_*j*_:

$$\begin{array}{ccccc} \underset{v_{j}\;\in \;R}{\text{maximize}\;\text{/}\;\text{minimize}} & & v_{j} & & \\ \text{subject}\;\text{to} & & & & \\ & & c^{⊺}v\geq \alpha \cdot z^{*} & \\ & & \text{Sv}=0 & \\ & & l_{i}\leq v_{i}\leq u_{i}, & & i=1,\ldots ,n \end{array}$$

where α∈R,0≤α≤1 represents the fraction of the optimum value with respect to the FBA objective value to be considered. FVA allows the user to infer specific properties of the fluxes involved. For example, *essential* reactions have strictly positive or negative fluxes, whereas *blocked* reactions are constrained to have a flux value equal to zero.

Both FBA and FVA are today state-of-the-art tools to explore CBMs [13]. From a computational viewpoint, several algorithms are available to solve these optimization-based approaches (see section Solving Linear Optimization Problems).

#### Thermodynamic constraints metabolic networks

FBA and FVA utilize constraints derived from mass conservation laws; however, it is possible to exploit thermodynamic laws to derive constraints in order to obtain further insights into the behavior of a metabolic system [31, 34, 35]. In biochemical systems, each metabolite has an associated chemical potential *μ*_*i*_ (expressed in J.mol^−1^), which quantifies the potential to perform chemical work. Chemical potentials depend on metabolite concentration according to $\mu_{i}=\mu_{i}^{0}+\text{RT}\;ln\left(x_{i}/x_{i}^{0}\right)$, where *x*_*i*_ is the molar concentration, $x_{i}^{0}$ is the standard reference concentration (1 M) and *μ*^0^ is the standard chemical potential (dependent on temperature, pressure, and ionic strength); these are usually tabulated [36, 37]. For a reaction *j*, the stoichiometric sum of the chemical potentials of the metabolites involved is equal to the Gibbs energy of the reaction, *i.e.*, $\Delta_{r}G_{j}=\sum_{i}^{n}S_{ij}\;\mu_{i}$ where Δ_*r*_ *G*_*j*_ ≤ 0 for a spontaneous reaction. In the following, we note the Gibbs energy of reaction as a difference of potentials, *i.e.*, Δ*μ*_*j*_ ≐ Δ_*r*_ *G*_*j*_.

Under NESS conditions, the entropy balance implies that **Δ***μ*^⊺^**v**_*ς*_ = ***μ***^⊺^**v**_*ξ*_, where **v**_*ς*_ represents the internal portion of fluxes, **v**_*ξ*_ boundary fluxes, and **Δ***μ* and ***μ*** are vectors of components Δ*μ*_*j*_ and *μ*_*i*_, respectively. The term ***μ***^⊺^**v**_*ξ*_ represents the *chemical motive force* or *cmf* of the network, which accounts for energy related to boundary fluxes [31]. This equation may be interpreted as internal fluxes being driven by the consumption of external chemical potential.

The integration of such equations into general CBMs is not straightforward, as in most of applications, concentrations *x*_*i*_ are not known; therefore, these must be introduced as variables. As a result of non-linear expressions, CBM formulations using these constraints are generally more complex to solve [38–40].

#### Solving linear optimization problems

In general, optimization problems are aim at determining *f*(**v**) where **v** is usually required to satisfy constraints. Linear optimization problems (LPs) are a particular kind of optimization problem where both objective function and constraints may be expressed as linear functions of variables, *i.e.*, *max* *f* = **c**^⊺^**v**, **Av** = **b**; where **v** is a vector of variables, **c** is a row vector of *n* coefficients, **A** is a matrix of *n* columns and *m* rows, and **b** a column vector of *m* values. The solution space of LP problems are polyhedrons that are characterized by their extreme points.

The first algorithm to solve a LP, which was proposed in 1947 by Dantizg [41], was based on the fact that if the objective function has an optimum value in the feasible region, then it reaches this value in at least one of the extreme points. The algorithm begins its search in one vertex of the feasible region and then starts visiting adjoint vertexes until the objective function value cannot be improved. Currently, several solvers such as GUROBI [42] or GLPK are capable of solving LPs and other types of single objective problems (SOPs) efficiently.

### From single microorganisms to microbial ecosystems

**Fig 2:**
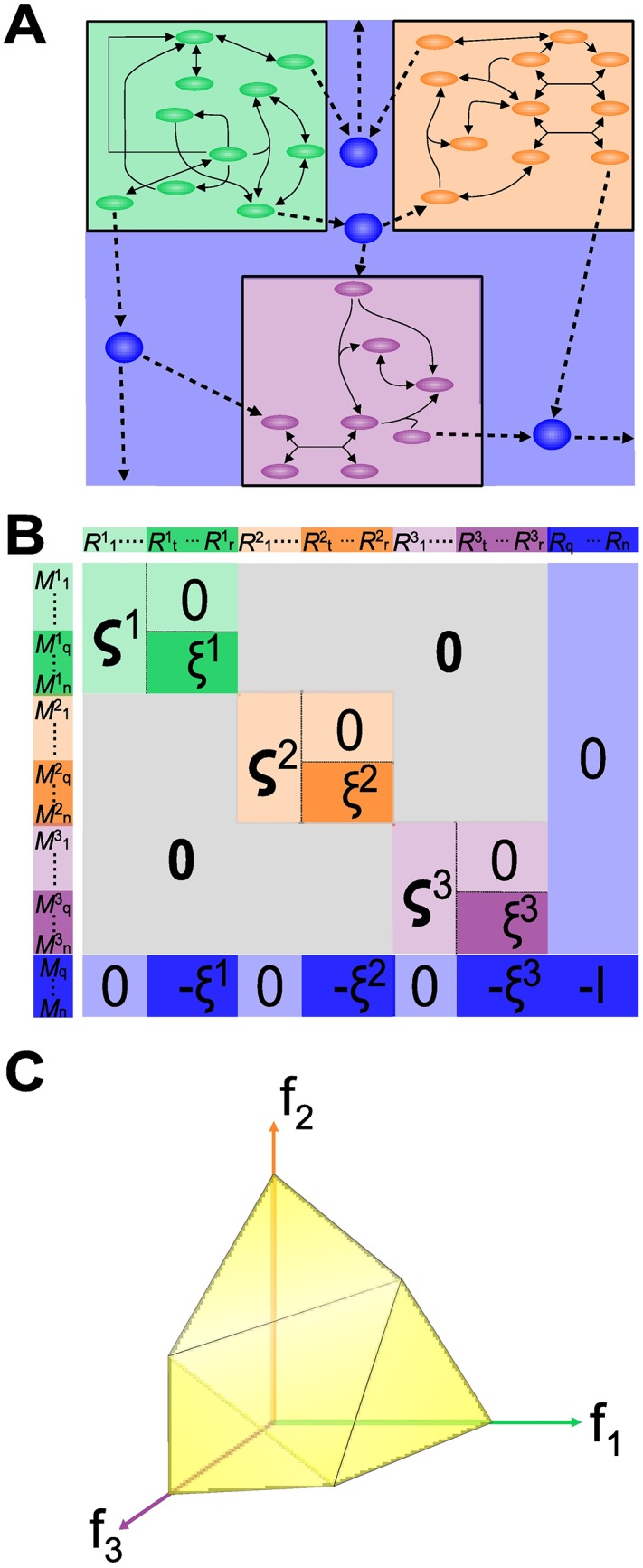
Illustration of microbial ecosystem CBM. For the sake of illustration, an ecosystem may be considered to comprise three microbial strains. (A) According to the metabolic model, each microorganism is considered a separate compartment, depicted here in green, orange, and purple. Metabolic networks are linked via an additional compartment, termed the “pool” (blue)), which sums up all external metabolites exchanged between organisms and the environment. (B) depicts the Stoichiometric Matrix **S**^*σ*^, where each compartment is colored accordingly, with their corresponding *ς* and *ξ* submatrices. (C) Pareto front. When performing an FBA for multiple organisms, a set of points known as the Pareto front (in yellow) is obtained. Objective functions **f**_**1**_, **f**_**2**_ and **f**_**3**_ define the “objective space”.

In order to model a microbial community, each strain is considered a single compartment [19, 25, 27] that shares metabolites with other strains (see Fig 2A). As the stoichiometric matrix of a single organism, the structure of the ecosystem is described by a stoichiometric matrix **S**^*σ*^, which is formed by the stoichiometric matrices of each single organism. Accordingly, for a community of *k* microorganisms, *k* metabolic models must be considered and represented by their corresponding stoichiometric matrices: **S**^*l*^, *l* = 1, …, *k*.

As shown in Fig 2B, matrices **S**^1^ to **S**^*k*^ are used to construct a diagonal block matrix. Each block is linked to a *pool compartment*, that mirrors exchange fluxes between each organism and the environment (−*ξ*^*l*^, for *l* = 1, …, *k* in Fig 2B). A set of exchange reactions *R*_*q*_ to *R*_*n*_ for metabolites *M*_*q*_ to *M*_*n*_ between the Pool and the external environment, is additionally set (bottom right in Fig 2B). Finally, as for single organisms, a steady state hypothesis restricts the solution set by adding a constraint **S**^**σ**^**v** = 0. Together with flux bound constraints *l*_*i*_ and *u*_*i*_, these constraints describe a solution flux space, as depicted in Fig 1C.

#### Multi objective flux balance analysis of a microbial ecosystem

Each compartment above corresponds to an organism with a specific objective function **c**_*k*_. Accordingly, the following multi-objective optimization problem, for analyzing flux balance conditions (MO-FBA), may be defined:

$$\begin{array}{ccc} \underset{v\;\in \;R^{\bar{n}}}{\text{maximize}}\; & \left(\begin{array}{c} f_{1}\\ \ldots \\ f_{k} \end{array}\right)=\left(\begin{array}{c} c_{1}^{⊺}v\\ \ldots \\ c_{k}^{⊺}v \end{array}\right) & \\ \text{subject}\;\text{to} & & \\ & S^{\sigma }v=0 & \\ & l_{i}\leq v_{i}\leq u_{i}\;\;i=1,\ldots ,\bar{n} \end{array}$$

where (*f*_1_, …, *f*_*k*_)^⊺^ are the objective functions of the *k* organisms and $\bar{n}$ is the total number of reactions (*i.e.*, the sum of reactions of each organism and exchange reactions from the pool compartment). The general class of MO-FBA problems is referred to as the *multi objective problems* (MOP) [29, 43]. Contrary to single objective problems, solution of MOPs is a set of vectors instead of a single value, producing a Pareto front (see section Solving Multi Objective Optimization Problems), defined in the objective space (Fig 2C). In our present formulation, all constraints and objective functions are linear, thereby resulting in a particular type of MOP known as the multi-objective linear problem (MOLP).

Interpretation of MO-FBA can be done in terms of growth rates and resources used to produce such growth. Indeed, if one of the members of the ecosystem decreases its growth rate, more resources are available for other members. According to their particular physiologies, they can use these new available resources to increase their own biomass. A guideline containing three ideal cases for two guilds is provided in S1 File.

#### Flux variability analysis of a microbial ecosystem

Given a particular point f* of the Pareto Front, the multiple optimal flux solutions that achieve the optimal objective values, as given by the Pareto optima f*, must be explored. To this end, we propose the use of the multi-objective FVA (MO-FVA) for multiple organisms, which may be considered a straightforward extension of FVA (see Flux Variability Analysis). Indeed, given a reaction *R*_*j*_ with $j=1,\ldots ,\bar{n}$, the range of the flux *v*_*j*_ may be determined by solving the following LPs:

$$\begin{array}{ccc} \underset{v_{j}\;\in \;R}{\text{maximize}\;\text{/}\;\text{minimize}}\;v_{j} & \\ \text{subject}\;\text{to} & & \\ & C^{⊺}v\geq \alpha \cdot f^{*} & \\ & S^{\sigma }v=0 & \\ & l_{i}\leq v_{i}\leq u_{i}\;\;i=1,\ldots ,\bar{n} \end{array}$$

where **C** is the matrix such as the column j corresponds to objective function **c**_*j*_, *i.e*, **C** is column defined as **C** = [**c**_1_, …, **c**_*k*_]. α∈R,0≤α≤1 is the fraction of the optima considered.

#### Thermodynamics analysis in the context of a microbial ecosystem

Biological systems are hypothesized to favor thermodynamic states where entropy production is maximal [44, 45]. To take into account this hypothesis, given a particular point f* of the front, we propose the following: First, a MO-FVA must be applied to determine *R*_*j*_ for each reaction, with $j=1,\ldots ,\bar{n}$ and the range [*a*_*j*_, *b*_*j*_] of the flux *v*_*j*_ near the Pareto optima f*. Next, the following optimization problem must be considered:

$$\begin{array}{ccccc} \underset{i\in \xi }{\text{maximize}} & & cmf=\sum \mu_{i}v_{i} & & \\ \text{subject}\;\text{to} & & & & \\ & & a_{i}\leq v_{i}\leq b_{i}, & & i\in \xi ,\\ & & \mu_{i}^{0}-{\text{dg}}_{i}\leq \mu_{i}\leq \mu_{i}^{0}+{\text{dg}}_{i}, & \end{array}$$

where *ξ* is the set of exchange reactions and ${\text{dg}}_{i}=RTln\left(x_{i}/x_{i}^{0}\right)$. As *cmf* is non-linear, optimization algorithms based on heuristics must be used in order to obtain a numerical solution to this problem (see Computational Procedures).

#### Solving multi objective optimization problems

In 1906, Vilfredo Pareto in his *Manuale di Economia Politica*, stated that, while (economic) optima have not been achieved, it is possible to increase the objective of an agent (*i.e.*, welfare) without decreasing that of another [46]. In the following, a formal definition of Pareto optima and efficient solutions is given [43] and approaches to solutions are discussed.

Let $X\subseteq R^{n}$ and $Y\subseteq R^{p}$ represent the flux space and objective space, respectively, where X is defined by the set of restrictions and Y:={y|y=f(x),x∈X}, with $f={\left(f_{1}\left(x\right),\ldots ,f_{p}\left(x\right)\right)}^{⊺}$ denoting the objective functions. If both X and Y are constructed using linear restrictions and linear objective functions, the MOP represents a MOLP.

A point y∈Y is a **Pareto optimum** if there is no $y^{*}\in Y$ such as $y_{j}^{*}\geq y_{j},j=1,\ldots ,p$ and **y** ≠ y*. Similarly, **y**^**w**^ is a **weak Pareto optimum** point if there is no y* such as $y_{j}^{*}>y_{j}^{w},j=1,\ldots ,p$. A point x∈X is an **efficient** solution if there is not a $x^{*}\in X$ such that **f**(x*) ≥ **f(x)**. A $x^{w}\in X$ is a **weak efficient** solution if there is no $x^{*}\in X$ such as **f**(x*) > **f**(**x**^**w**^). Therefore, a (weak) Pareto optimum is the image of a (weak) efficient solution. Note that all efficient solutions are also weakly efficient solutions but no vice-versa. The collection of Pareto optimal points is termed **Pareto Front**.

Approaches for solving MOPs have been reviewed, for example, by [43] and [47]. Traditional approaches makes use of “scalarization techniques”, that involve the transformation of the MOP into a SOP by using a real-valued scalar function of the objective functions. Solution approaches using scalarization techniques aim to find the set of (weak) efficient solutions $x^{*}\in X$.

The most well known approach is the “weighted sum approach”, wherein the weighted sum of the objective functions is optimized, *i.e.*, max ∑*λ*_*k*_ *f*_*k*_(**x**), where x∈X and $\lambda \in R^{p}$ is a given weight vector with components *λ*_*k*_ ≥ 0 and at least one *λ*_*k*_ > 0. If x* is a solution of this SOP then x* is an efficient solution of the MOP. Furthermore, if the MOP is convex, the inverse is also true.

Another commonly used approach is the “*ϵ*-constraint method”, where only one objective function is retained as the objective and the remaining objective functions are used to introduce new constraints. Then, the j-th *ϵ*-constraint problem is as follows: max *f*_*j*_(**x**), subject to *f*_*i*_(**x**) ≥ *ϵ*_*i*_, *i* ≠ *j* and x∈X. If x* is a solution of this SOP, then x* is a weak efficient solution of the MOP.

Not all approaches rely on scalarization: for MOLPs, a set of algorithms describing the shape of the image of efficient points, $Y_{E}:=\left\{\text{Cx}\;|\;x\;\text{is}\;\text{efficient}\right\}$, referred to as “outer approximation” or “Benson type” algorithms, have been described [48–51]. Generally speaking, these type of algorithms calculate Y and identify their vertices, which correspond to Pareto optimal points; additionally, despite their names, these algorithms provide exact solutions. BENSOLVE [52], a solver based on these approaches, computes a set of directions and points describing the image of the efficient points.

#### Existing CBM approaches for communities

The various approaches to studying microbial communities have been recently reviewed by Biggs *et al.* [21] and Perez-Garcia *et al.* [22]. Among the methods reviewed, OptCom most closely resembles the approach presented here, in that each member of the community is considered to maximize its own biomass. OptCom is based on bi-level optimization, where an “outer” maximization problem represents the whole community and each member of the community is represented by a “inner” optimization problem. Inner optimization problems are solved using the primal-dual theorem, which transforms the whole bi-level formulation into a non-convex single-objective form [25]. A second approach that combines compartments and FBA, known as community flux balanced analysis, advocates the application of a *“balanced growth”* hypothesis, wherein each compartment grows at the same rate. Furthermore, this approach considers the biomass fraction of each member of the community. In general, the approach is non-linear, although it may be made linear by fixing biomass fractions and solving the corresponding FBA. Then, optimal solutions for various combinations of biomass fractions may be explored [27]. For illustration purposes, the application of our approach to the analysis of a microbial ecosystem is discussed below.

### Case study: Hot spring mat

**Fig 3:**
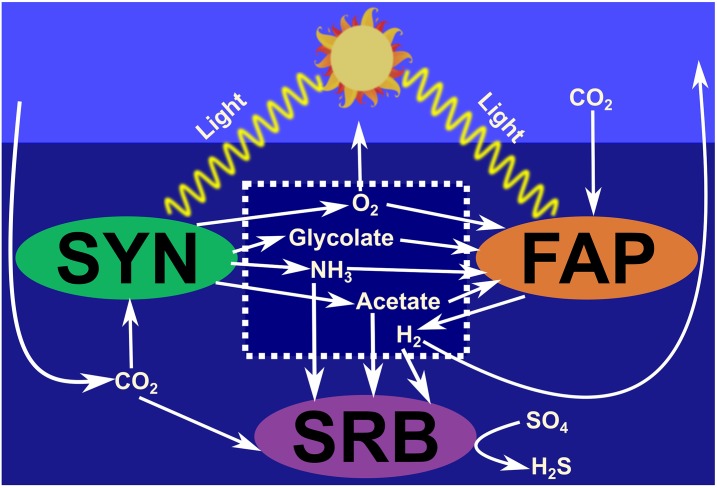
Day Model of the Hot Spring Mat Community. The model comprises three guilds of microorganisms of the SYN, FAP, and SRB phenotypes. Organics acids produced by SYN may be utilized by FAP and SRB. FAP is capable of fixing carbon by anoxygenic photosynthesis. Under anoxygenic fermentation conditions, FAP is additionally capable of producing hydrogen, which, in turn, may be used by SRB.

In order to illustrate the application of the present approach, we modeled the microbial ecosystem of hot spring microbial mats [33]. Briefly, this ecosystem is composed of three *guilds*, representing three commonly found phenotypes: *Synechococcus spp.* (SYN), *Chloroflexus spp.* and *Roseiflexus spp.* (FAP) sulfate-reducing bacteria (SRB). SYN is a primary producer that fixes carbon and nitrogen for further utilization by other strains. The use of these guilds allows simplification of the ecological diversity while capturing essential metabolite-exchange relationships. Under light conditions, the major fate of nutrients involves assimilation into cells [53]; therefore, most of the overall system growth occurs during the daytime. As growth rates are related to biovolumes, predictions may be compared with relative abundance data. Therefore, we will focus on the daytime model as described in [33] (Fig 3), assuming a simplified night-time behavior, as described below.

Using the available compartment model of this system, as described in [33], we performed a manual curation (*i.e.*, balancing equations and including intermediate reactions) using METACYC [54]. Model equivalent reactions in [33] are provided in S2 File. Nitrogen fixation has been shown to take place at night and in the early morning [55, 56]; therefore, a nitrate assimilation mechanism for SYN was included and considered as functional. Finally, biomass coefficients of each guild were scaled to match 1 (h^−1^) as maximal growth rate [57].

Glycolate is produced by the use of O_2_ instead of CO_2_ by the Rubisco enzyme; the flux ratio between the use of O_2_ and CO_2_ varies between 0.03 and 0.07. This restriction was included linearly in the model by fixing a ratio of 0.03 between SYN reactions RXN-961 and RIBULOSE-BISPHOSPHATE-CARBOXYLASE-RXN during all calculations, under the hypothesis that the system is in anaerobic state.

Excess photosynthate producing during the day is stored as polyglucose (PG) by SYN. PG is fermented at night, producing several organic acids that accumulate in the media and are integrated as biomass mostly under light conditions [53, 58]. In order to capture this behavior in the daytime model, PG was not allowed to accumulate; therefore, the excess photosynthesis activity is redirected through acetate production. Accordingly, in our model, acetate is interpreted as equivalent to several forms of reduced carbon.

For each of the exchanged metabolites, standard Gibbs energies for biological conditions were obtained from [37], using calculations from [36]. Values used are found in S2 File. For the pseudo-compound *hv* (representing photons), a standard chemical potential was estimated based on glucose synthesis from CO_2_: 6CO_2_+6H_2_O $\overset{48\;\mathrm{hv}}{\to }$ C_6_H_12_O_6_. The assumption that this reaction approaches equilibrium at standard biological conditions (*i.e.*, Δ*μ* = 0) implies that *μ*_*hv*_ = 68.6 kJ.mol^−1^ (S2 File). The metabolite concentration was allowed to vary between 10^3^ and 10^−3^ M, and therefore chemical potential equals $\mu_{i}=\mu_{i}^{0}\pm \text{dg}$, where dg = *RTln*(10^3^) ≈ 20 (kJ.mol^−1^) for T = 75°Celsius. For water and *hv*, concentrations were considered as fixed at 1 M, implying dg_H_2_O_ = dg_hv_ = 0.

### Computational procedures

For each guild, a metabolic model was built in MATLAB and an ecosystem stoichiometric matrix **S**^*σ*^ was constructed, as described above. MO-FBA was carried out using BENSOLVE [52]. In order to analyze nitrogen and carbon fluxes through MO-FBA results, a MO-FVA was performed using GUROBI [42] through Python interface over a mesh of 5 151 equally distributed points in the Pareto surface at 90% fraction of optimum. Then, we subdivided the Pareto surface into 225 similar regions; for each of these regions, we calculated their maximum (as well as their minimum) as the average of MO-FVA maxima of mesh points contained (this procedure was repeated for the minima). Thermodynamics calculations were performed over the same mesh as the MO-FVA using a truncated Newton conjugate algorithm [59] contained in scipy optimization module. Heatmaps and surface illustrations were generated using matplotlib [60] with *ad-hoc* scripts.

From methods discussed in Biggs *et al.* [21] and Perez-Garcia *et al.* [22], OptCom [25] was chosen for comparison, as this method resembles the approach applied to the present work. We applied OptCom and Descriptive OptCom to the hot spring mat model, as follows: first, 11 points were calculated using OptCom, as described by [25], each with a different upper boundary value for SYN biomass; these values ranged from 1.0 to 0.0 with a step of 0.1 (i.e. 1.0, 0.9, 0.8, …, 0.0). Second, Descriptive OptCom was applied three times using SYN to FAP ratios of 1.5, 2.5, and 3.5, respectively. All programs were written in GAMS language and solved using BARON [61] through the NEOS Server [62–64].

All scripts are available in https://gitlab.univ-nantes.fr/mbudinich/MultiObjective-FBA-FVA

## Results

### Biomass distribution as relative microbial strain abundance

**Fig 4:**
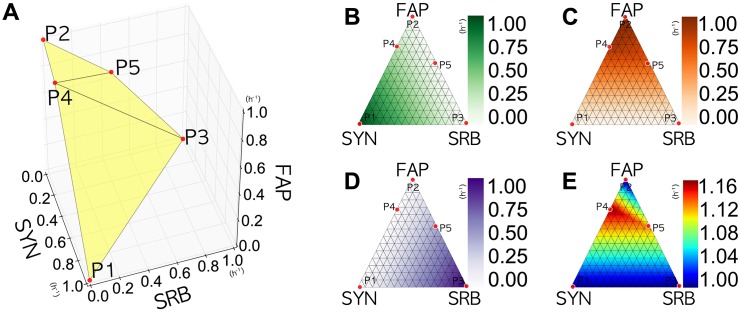
3D and 2D Projections of Pareto Front. (A) shows a 3D Pareto front, in yellow, describing the maximal growth rates of SYN, FAP, and SRB (in terms of units per hour, h^−1^), when considered as a system. It is evident that a decrease in the growth rate of one organism results in an increase in that of the other two, but not necessarily in equal proportions (see S1 Video for an animated view). The sum of the growth rates of all the guilds in P4 and P5 was 1.16 (h^−1^) and 1.11 (h^−1^), respectively. In (B), (C), (D), and (E), the Pareto front was projected onto the triangular surface formed by P1, P2, and P3. (B), (C), and (D) shows the respective growth rates for SYN, FAP, and SRB, respectively. (E) shows the sum of the three growth rates, which represent the total biomass of the ecosystem.

SYN, SRB, and FAP growth rates are represented in a 3-dimensional space, in each axis, respectively, in Fig 4A. MO-FBA solutions are described as a Pareto front, representing a surface with five extreme points of biomass growth: (1, 0, 0), (0, 1, 0), (0, 0, 1); the points corresponding to the maximal growth rates of each guild, and points (0.27, 0.00, 0.89) and (0.00, 0.46, 0.65). In the following, these points are designated P1, P2, P3, P4, and P5, respectively. For clarity, this Pareto front is then projected in a two-dimensional space. Therefore, over a triangular surface defined by P1, P2, and P3, heatmaps were produced using the values for the growth rate of SYN, FAP, SRB, as well as their sum, to depict the overall microbial abundance (Fig 4B–4E, respectively). Each vertex of the triangle represents the maximal growth rate of a guild, while its opposing side represents a zero growth rate for that guild.

The results show that when each guild grows at its maximal rate, no biomass is produced by the other guilds. The sum of the growth rates is always minimal in vertices (blue areas in Fig 4E). As the growth rates may be directly related to biovolumes [33], red to yellow areas in Fig 4E represent regions where most of the total biomass of the ecosystem is present. Notably, these regions correspond to guilds growing at sub-optimally rates.

### Nitrogen and carbon fluxes between microbial guilds

**Fig 5:**
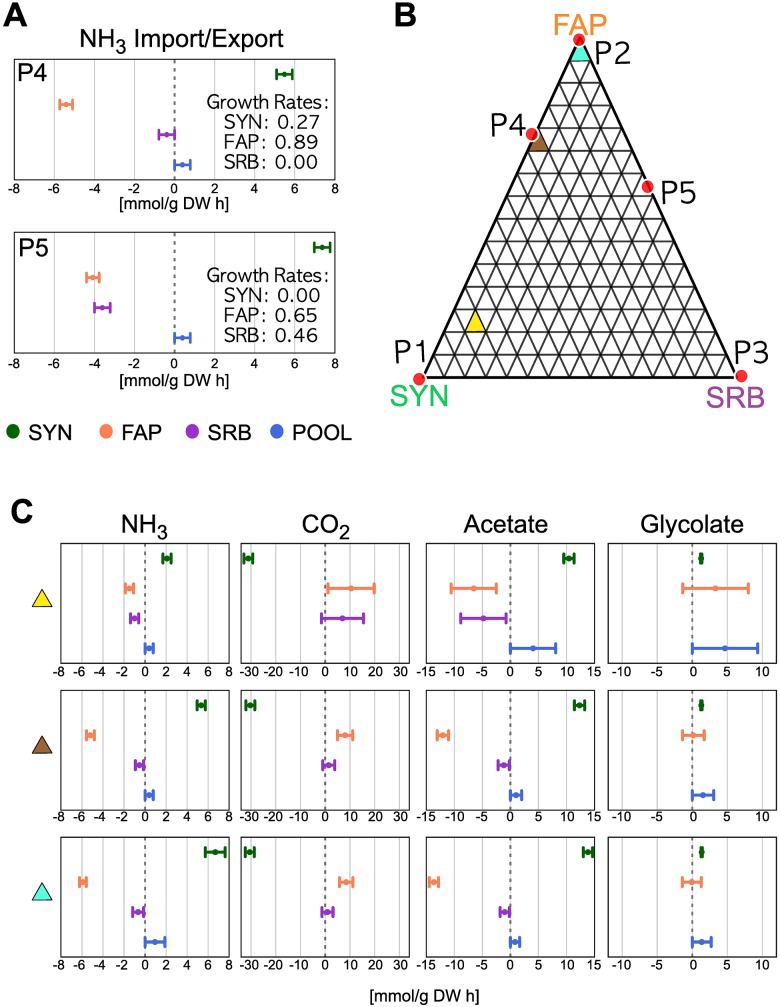
Multi Objective FVA. (A) shows NH_3_ maximal and minimal fluxes for SYN, FAP, SRB, and pool compartments (green, yellow, purple, and blue respectively) for extreme points P4 and P5. The export of NH_3_ by SYN is correlated with a drop in their growth rate; similarly, increases in NH_3_ intake are correlated with increases in the growth rates of FAP and SRB. (B) Three sections selected for the illustration of MO-FVA; (C) Mean values of the minimal and maximal fluxes over selected sections of NH_3_, CO_2_, acetate, and glycolate (columns) for each section (rows).

Multi-objective FVA was performed in the P4 and P5 regions to explore NH_3_ import and export fluxes between guilds (Fig 5A, upper and lower panel, respectively). Notably, the growth rate of each strain was found to be related to the use of ammonia; the SYN guild re-oxidized ferredoxins, which were reduced in the photosynthetic reactions, via nitrate assimilation reactions, thereby promoting permanent ammonia production. When growing sub-optimally, NH_3_ that is not used to build biomass is excreted. This point is emphasized in Fig 5A, where both maximal and minimal reaction rates are strictly positive for SYN, resulting in an export to the pool.

Nitrogen uptake by FAP and SRB occurs solely from ammonia that is available in the pool compartment; therefore, these strains compete for its intake. When SRB is not growing (superior panel in Fig 5A), excess of NH_3_ is taken up mainly by FAP (both minima and maxima are negative, implying an intake from the pool). Small amounts that are not taken up by FAP may be either taken up by SRB (maximal rate value is null and minimal rate negative, which depicts a possible import) or excreted to the external environment (pool maximal rate value is positive and minimal rate value is null, which depicts a possible export to the media). When SRB is growing (inferior panel of Fig 5A), the uptake rate of ammonia by SRB and FAP is similar, with no export to the external media.

In order to analyze the relationships between the growth rate of each strain and nitrogen- or carbon-related fluxes, we performed a MO-FVA as described in Computational Procedures, focusing on exchange reactions. For the purpose of illustration, we highlighted three sections from 225 calculated, as shown in Fig 5B. These regions were chosen to depict the theoretical interplay between SYN and FAP when the growth rate of SRB is low [65]. Flux variability of exchange fluxes for these regions is shown in Fig 5C (see S1 Fig for an alternative representation and S2 to S5 Figs for a complete MO-FVA for ammonia, acetate, carbon dioxide and glycolate fluxes).

For NH_3_ exchange reactions, high growth rates of SYN are related to lower levels of ammonia export, which represents a limiting factor for FAP and SRB growth rates. This results in the two strains competing for its use (S2 Fig). Fig 5C shows that most of the ammonia produced by SYN is captured by FAP, while a small proportion is taken up by SRB. Ammonia that is not captured is released into the pool.

SYN consumes approximately the same amount of CO_2_ under all relative abundance conditions (see second column in Fig 5C and S4 Fig), indicating that carbon compounds are involved in reactions that serve functions other than biomass synthesis. Acetate intake by FAP is less restrained at low growth rates of SYN than at high growth rates (see Fig 5C and S3 Fig).

The present results additionally emphasize that FAP and SRB produce relatively small amounts of CO_2_ at low growth rates. However, when the growth rate of FAP increases, the maximal excretion of CO_2_ reduces, whereas its minimal excretion increases; these data indicate the theoretical efficiency of carbon management, as experimentally reported by [53]. Glycolate metabolism by FAP appears to be reversible as its minimal flux is negative (*i.e.*, intake) while its maximal flux is positive (*i.e.*, excretion), implying that intake or excretion by FAP is related to the relative abundance of other strains (see Fig 5C and S5 Fig for details).

### Chemical potentials drive community growth rates

**Fig 6:**
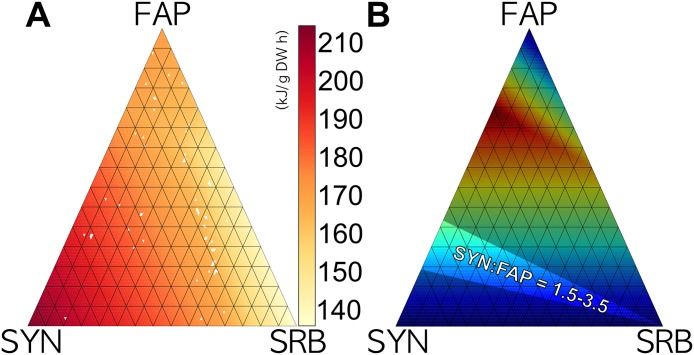
Thermodynamics in the Pareto front. (A) Description of the chemical motive force (kJ.gr^−1^.DW^−1^.h^−1^) for each point of the Pareto front; red regions indicate thermodynamically favored growth rates, while the points where the solver does not reach the optimal criteria are shown in white. The obtained surface appears smooth, without sudden changes in neighboring values. (B) Description of the overall community biomass distribution based on the growth rate of each strain, with a particular emphasis on regions supported by experimental measurements showing a SYN: FAP ratio of between 1.5 and 3.5.

As discussed previously, the direct integration of thermodynamic constraints into MO-FBA and MO-FVA formulations is complex. Instead, we used the thermodynamic optimization problem stated in as a post-treatment analysis. Considering fluxes as computed by MO-FVA in 5 151 points of Pareto front (as a result of which growth rates are also determined), we estimated the corresponding maximal *cmf* for each point (Fig 6A).

Results show that higher *cmf* is associated with SYN growing at its optimal rate. Lower *cmf* rates are related to a higher growth rate of SRB, whereas the impact of the growth rate of FAP on the value of *cmf* appears to be lower than that of SRB.

Given that all surface showed positive values, all regions are feasible from a thermodynamic viewpoint. Under the hypothesis that a biological system prefers configurations in which entropy production is maximal, it is expected that an ecosystem would favor growth rates with higher *cmf* (redder areas in Fig 6A), predicting higher SYN growth rates. This prediction is consistent with *in vivo* field measurements of SYN: FAP relative abundance ratios in the range of 1.5 and 3.5, with a low presence of SRB [33, 65], as shown in Fig 6B.

### Comparison with previous approaches

We compared growth rates and flux predictions of MO-FBA and MO-FVA with those obtained by a comparable approach (OptCom [25]), as described in Computational Procedures. Predictions obtained were mapped as points in the Pareto front (S6 Fig). Values of growth rates, as well as their corresponding flux values for NH_3_, acetate, glycogen, and CO_2_, are described in S2 File. As expected, all points calculated using the OptCom approach were included in the Pareto front calculated by MO-FBA (S6 Fig). Furthermore, all flux predictions for NH_3_, acetate, glycogen, and CO_2_ fall into the range predicted by MO-FVA. Without constraining SYN biomass (point O1), OptCom does not reach the maximal biomass optimum. However, when SYN biomass is increasingly constrained (points O2 to O11), the total biomass increases. This suggests the existence of local optima in the OptCom general formulation for this model.

The composition of a community that function in a constant environment can be also assessed using the approaches proposed in [27] and [28]. Here, we focus on modeling the composition of a community in a changing medium where the considered organisms could grow not necessarily with the same growth rate

## Discussion

As reported in previous studies, in particular [25], we extended state-of-the-art systems biology constraint-based approaches to the modeling of microbial ecosystems, by considering a multi-objective optimization framework. Within the ecosystem, each microorganism, with its own objective function, represents a building block that interacts with others via the exchange metabolites. Furthermore, the genomic knowledge of each microorganism is integrated as a set of metabolic constraints. The main advantage is represented by the capture of trade-offs on objectives and metabolite exchange between members of the ecosystem. While previous works report topological analyses that focus on pathways that promote cross-feeding between strains (see [66, 67] for example), this study quantifies fluxes through these pathways as well as their effect in objective functions, thereby representing a major step towards automatically producing trait-based models. Through the application of MO-FBA, we emphasize a full description of the Pareto front that captures trade-offs in the optimal values of the objective function of each microorganism. Additionally, we introduced MO-FVA as a tool for the analysis of exchange fluxes between members of the community. These fluxes help to characterize the optimal behavior of microorganisms, providing insights into the theoretical relative abundances (*i.e.*, a proxy for microbial diversity) and corresponding nutrients usage, that are based on *omics* descriptions.

Unlike previous works that consider multiple objectives, our approach does not rely either on assumptions about ecosystem behaviors, such as maximization of the total ecosystem biomass, ([25, 26]) nor on the balanced growth ([27, 28]) of microbial strains involved. Instead, we propose to describe all optimal solutions in the sense of Pareto in the objective space. This approach provides several advantages: firstly, it includes any solution for a system objective function expressed as a weighted sum of each compartment objective function (see [43] and section Solving Multi Objective Optimization Problems). Therefore, it comprises all solutions proposed by OptCom as system objectives for microbial communities [25]. Secondly, no additional complementary restrictions are required to focus on given solutions, i.e., imposing an equal growth rate for all members, as proposed by Kandelwal et al. [27]. This restriction remains valid for controlled microbial ecosystems. Third, the set of constraints remains linear, which allows a description of the Pareto front for realistic ecosystems. In [25] and [26], formulations are, in general, non-convex; in [27], the stated general optimization problem is non-linear. However, in order to solve MOLPs, a series of LPs must be solved for which exact algorithms are fast, thereby reducing computational complexity. Note herein that the last two points are mandatory to model natural ecosystems that are by definition composed of a large number of microbial strains and mostly unconstrained.

For illustration purposes, we applied MO-FBA to the daytime part of the diurnal cycle of the microbial hot spring mat system [33]. As most biomass fixation occurs during the day phase [53], we assumed that daytime growth rates dominate overall ecosystem rates. Results show that the maximal total biomass growth rate is achieved when each guild grows at a rate below its theoretical maximum, which may, based on genomic knowledge, be interpreted as an altruistic behavior. Mechanistically, when guilds make resources available to others, they lower their objective value by a certain proportion, based on metabolic pathways used to synthesize those resources and their biomass function. Conversely, the use of new available resources increases the value of the objective functions of the other guilds. Therefore, the growth rate of the global maximal ecosystem, which was designated P4 in our case study, should correspond to the optimal resource allocation scenario from the ecosystem viewpoint. P4 also corresponds to the optimal solution to maximal ecosystem biomass [25].

MO-FVA results show that nitrogen flux is correlated to growth rates, and that the three guilds compete for their usage. In contrast, CO_2_ consumption and glycolyte and acetate production by SYN do not seem to be correlated with its growth rate, indicating that these processes are not carbon-limited. Reduced carbon, represented by acetate, appears as being the main carbon flux in the system for FAP and SRB, and becomes a limiting nutrient for FAP at high growth rates. This result is consistent with those of [53] and [58], in which a high proportion of reduced carbon was shown to be assimilated by FAP.

By coupling MO-FVA results with chemical potentials, we were able to analyze thermodynamic constraints and study favored conditions of the Pareto front by comparing their respective maxima *cmf*. We observed that the SYN: FAP ratio, predicted using this criteria, is closer to the 1.5 to 3.5 value observed in field measurements. Thermodynamic considerations underline relative strain growth rates, or microbial diversities, that are more favorable from an energetic viewpoint, which indicates that an ecosystem behaves according to two different objectives: maximal biomass production and maximization of *cmf*, corroborating previous systems biology studies that advocate the use of distinct concurrent objectives to predict *Escherichia coli* metabolic behaviors [68]. In both cases, observations were possible by general investigation of the Pareto front.

Nevertheless, further refinement of the thermodynamic calculations is warranted. In particular, the calculation of *cmf* does not consider biomass concentration; this may be overcome by considering community fractions as proposed in [27] and [28]. Furthermore, in the current model, biomass generation does not affect the overall ecosystem entropy; however, on an intuitive basis, a larger amount of biomass should increase an entropy term, in terms of Gibbs energy, as a result of mass dispersion [69], thereby affecting *cmf* evaluation. These considerations are out of the scope of the present work; however, they but raise interesting perspectives.

Despite the above limitations, we consider the present form of the modeling approach as fruitful guidance to gain qualitative as well as quantitative data for the metabolic interplay between various species in an ecosystem. This method paves the way for improved contextualization of other -omics datasets in microbial ecology by providing a mechanistic description of species co-occurrence *via* analysis of their metabolic interactions.

## Supporting information

S1 File Guidelines for interpreting MO-FBA results. (PDF)

S2 File Metabolic Model of Hot Spring Community. A Stoichiometric Matrix of each guild used, along with thermodynamic data considered.
(XLSX)

S1 Video Animated 3D version of Pareto front. (MP4)

S1 Fig Alternative MO-FVA illustration of Fig 5. The convention used is the same for S2–S5 Figs.
(EPS)

S2 Fig MO-FVA for NH_3_ exchange fluxes between SYN, FAP, and SRB. (PNG)

S3 Fig MO-FVA for acetate exchange fluxes between SYN, FAP, and SRB. (PNG)

S4 Fig MO-FVA for CO_2_ exchange fluxes between SYN, FAP, and SRB. (PNG)

S5 Fig MO-FVA for glycolate exchange fluxes between SYN, FAP, and SRB. (PNG)

S6 Fig OptCom and Descriptive OptCom results mapped in the Pareto front. (PNG)
