# Supplementary material for: A multi-objective constraint-based approach for modeling genome-scale microbial ecosystems
Source: PLoS One. 2017 Feb 10;12(2):e0171744. doi: 10.1371/journal.pone.0171744 (PMC5302800; doi:10.1371/journal.pone.0171744)
Supplement: S1 File — (PDF) [file pone.0171744.s001.pdf]

GUIDELINES FOR UNDERSTANDING MO-FBA RESULTS

1. Introduction

In the following document, we describe a toy example of MO-FBA using two microorganisms, in order to give some insights for more realistic cases. MATLAB script `GenerateExamples.m` generates the corresponding case studies. (https://gitlab.univ-nantes.fr/mbudinich/MultiObjective-FBA-FVA)

In the following, we consider a microorganism such as it physiology consists only in the production of Biomass BM from compounds A and B, *i.e.,* biomass reaction correspond to: $\mathrm{a\,A} + \mathrm{b\,B} \longrightarrow \mathrm{BM}$. In addition, we will consider that this hypothetical microorganism has transporters for A and B. Furthermore, BM is also considered to be exported outside the organism (see Figure 1).

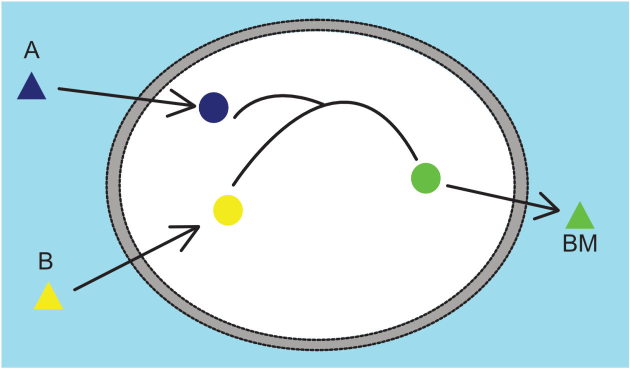

Figure 1. Hypothetical Microorganism. Biomass is created using A and B, which are imported from the media. BM are excreted from system.

Next, we will consider two strains of such microorganisms, named Org1 and Org2, with objective functions named f_1 and f_2, respectively. Applying the modeling protocol described in the main text, the pool compartment is constructed. Finally, three illustrative cases will be studied (see `GenerateExamples.m` for details in the implementation):

Case I : Org1 and Org2 are identical, with $\mathrm{a} = \mathrm{b} = 1$.
Case II : Org1 is more efficient in biomass construction, *i.e.*, $\mathrm{a} = \mathrm{b} = 1/2$ for Org1. Org2 is kept with $\mathrm{a} = \mathrm{b} = 1$.
Case III : Org1 and Org2 have different resource usage, *i.e.*, $\mathrm{a} = 1$, $\mathrm{b} = 1/2$ for Org1 and $\mathrm{a} = 1/2$, $\mathrm{b} = 1$

2. Results

A MO-FBA was run for these three cases. Results of Pareto Front calculation are summarized in Figure 2. Case I is pretty straightforward, the line observed is what one would expect if a perfect competition is occurring between both strains. Resources that are released with each decrement in growth rate from one organism are used in the same way by the other strain.

In Case II, where Org1 is more efficient, resources released when Org2 decreases its growth rate and a higher increment Org1 growth rate per unit of resources is obtained.

Finally, Case III shows two regions reflecting different limiting resources. When Org2 is near its maximal growth, it consumes available B, while A is still present. When growth rate of Org2 decreases, B is available, and used more efficiently by Org1, incrementing its growth rate. When both Org1 and Org2 growth rates are at (2/3,2/3), an optimal global resource assignation occurs: 2 thirds of A is used by Org1 and 2 thirds of B by Org2. For growth rates over 2/3, A becomes limitant for Org1; as Org2 decreases their growth rate, more A is available but, as Org1 is less efficient in its use, increment in its growth rate is less significant.

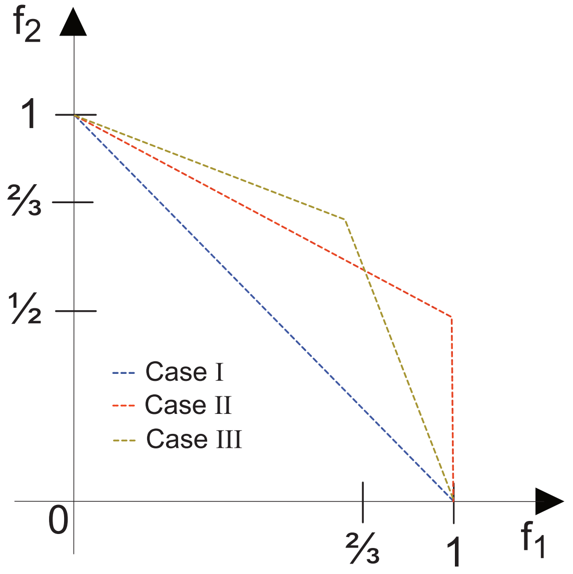

Figure 2. Pareto Front for the three analyzed cases
