# Supplementary figures and images for: A multi-objective constraint-based approach for modeling genome-scale microbial ecosystems
Source: PLoS One. 2017 Feb 10;12(2):e0171744. doi: 10.1371/journal.pone.0171744 (PMC5302800; doi:10.1371/journal.pone.0171744)

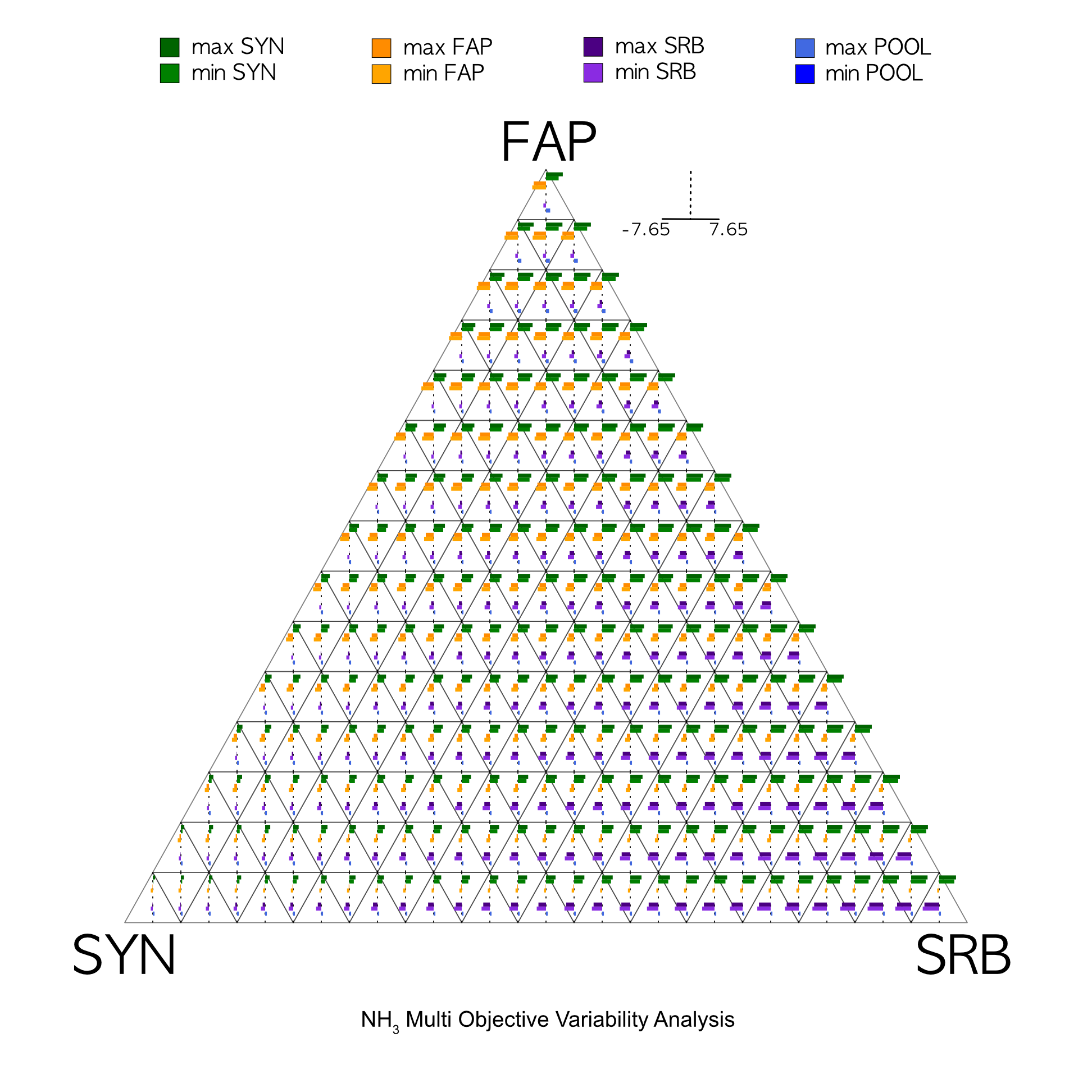

Supplement: S2 Fig — (PNG) [file pone.0171744.s005.png]

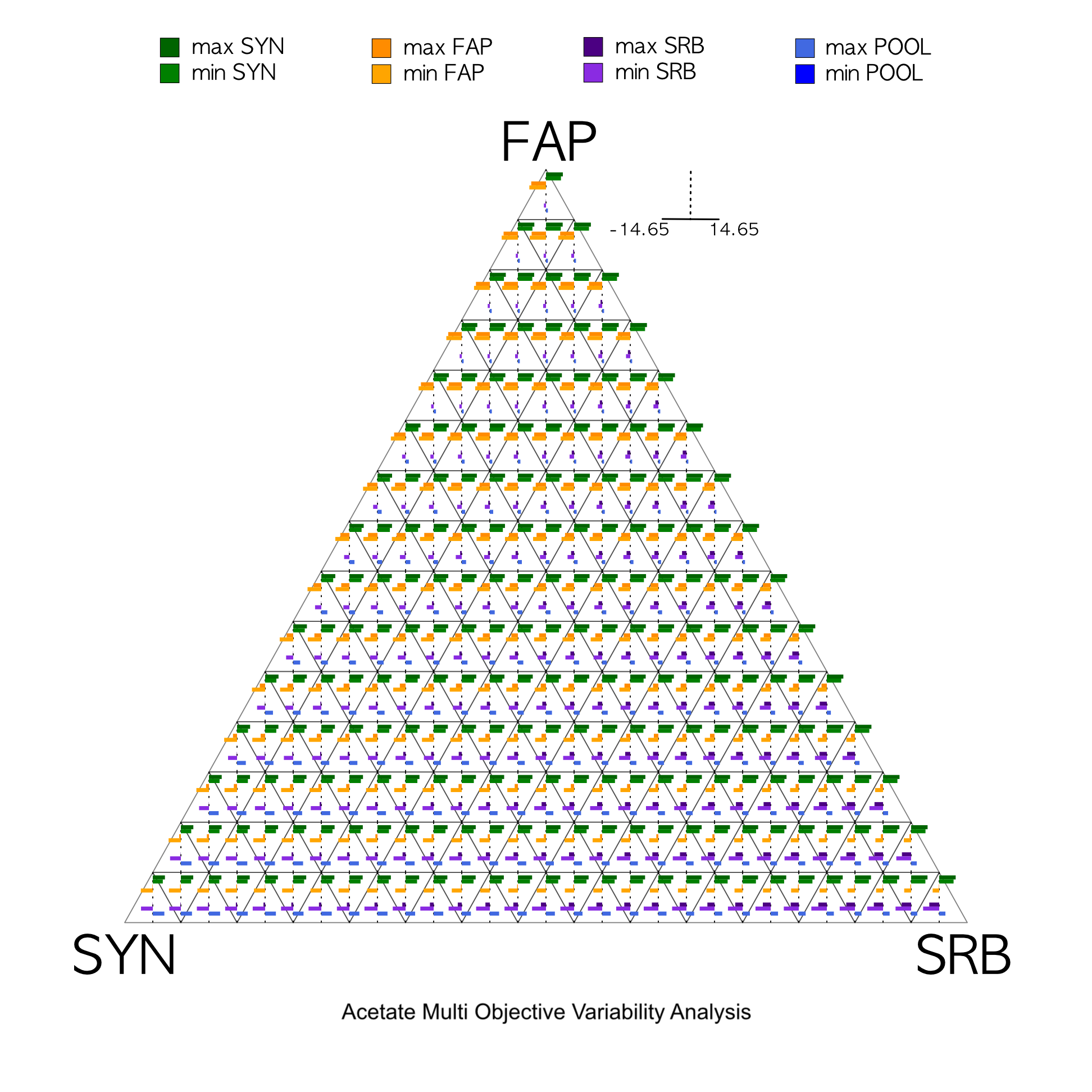

Supplement: S3 Fig — (PNG) [file pone.0171744.s006.png]

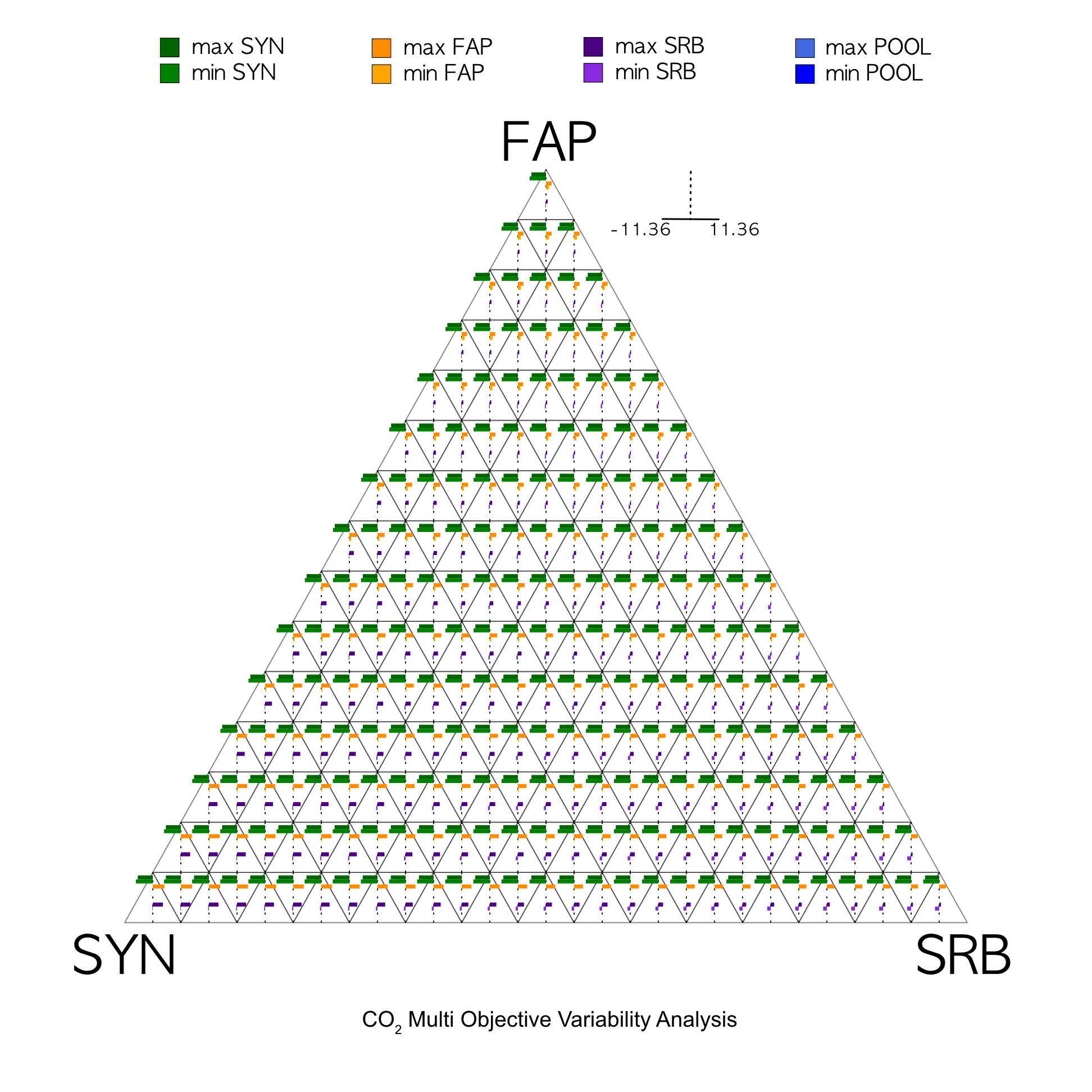

Supplement: S4 Fig — (PNG) [file pone.0171744.s007.png]

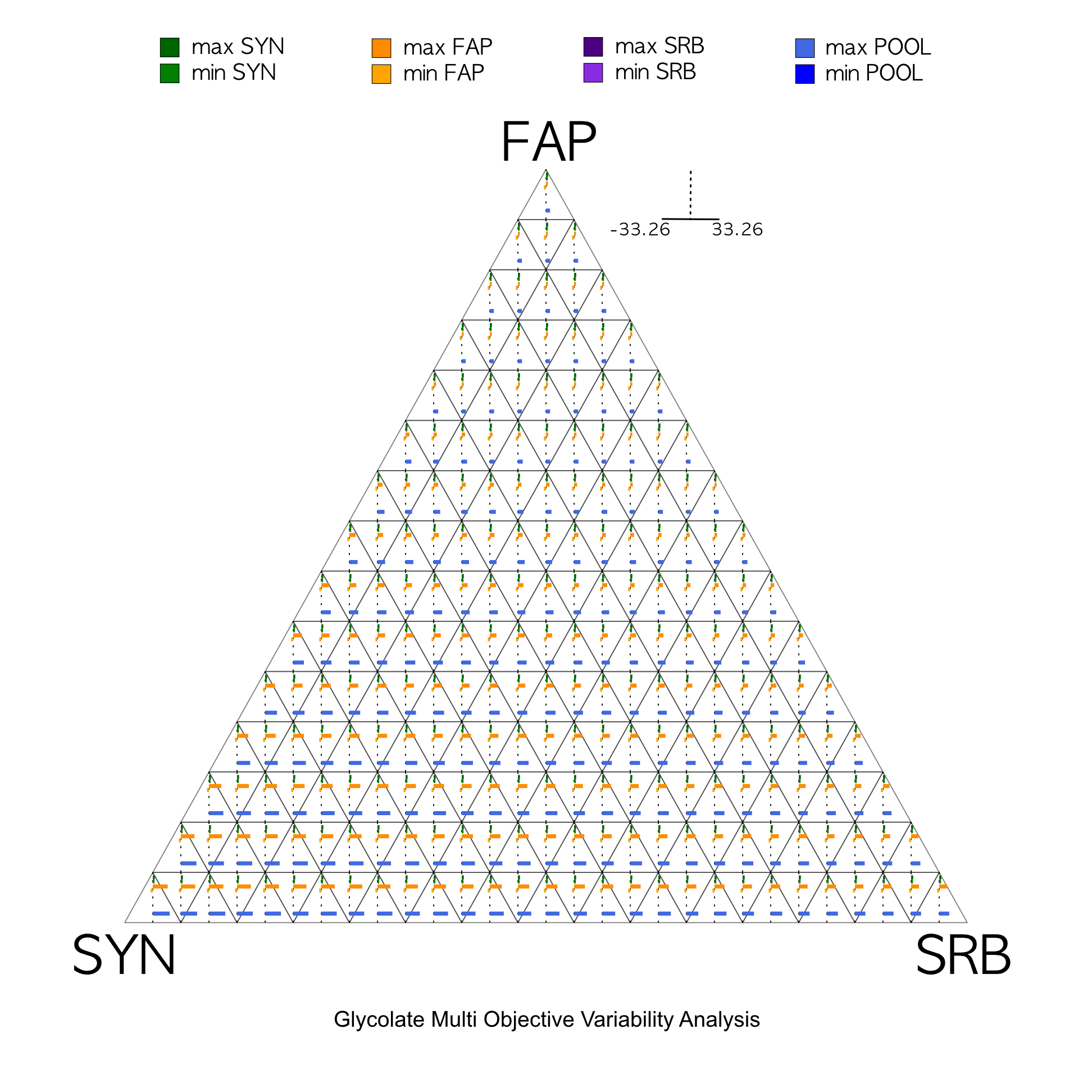

Supplement: S5 Fig — (PNG) [file pone.0171744.s008.png]

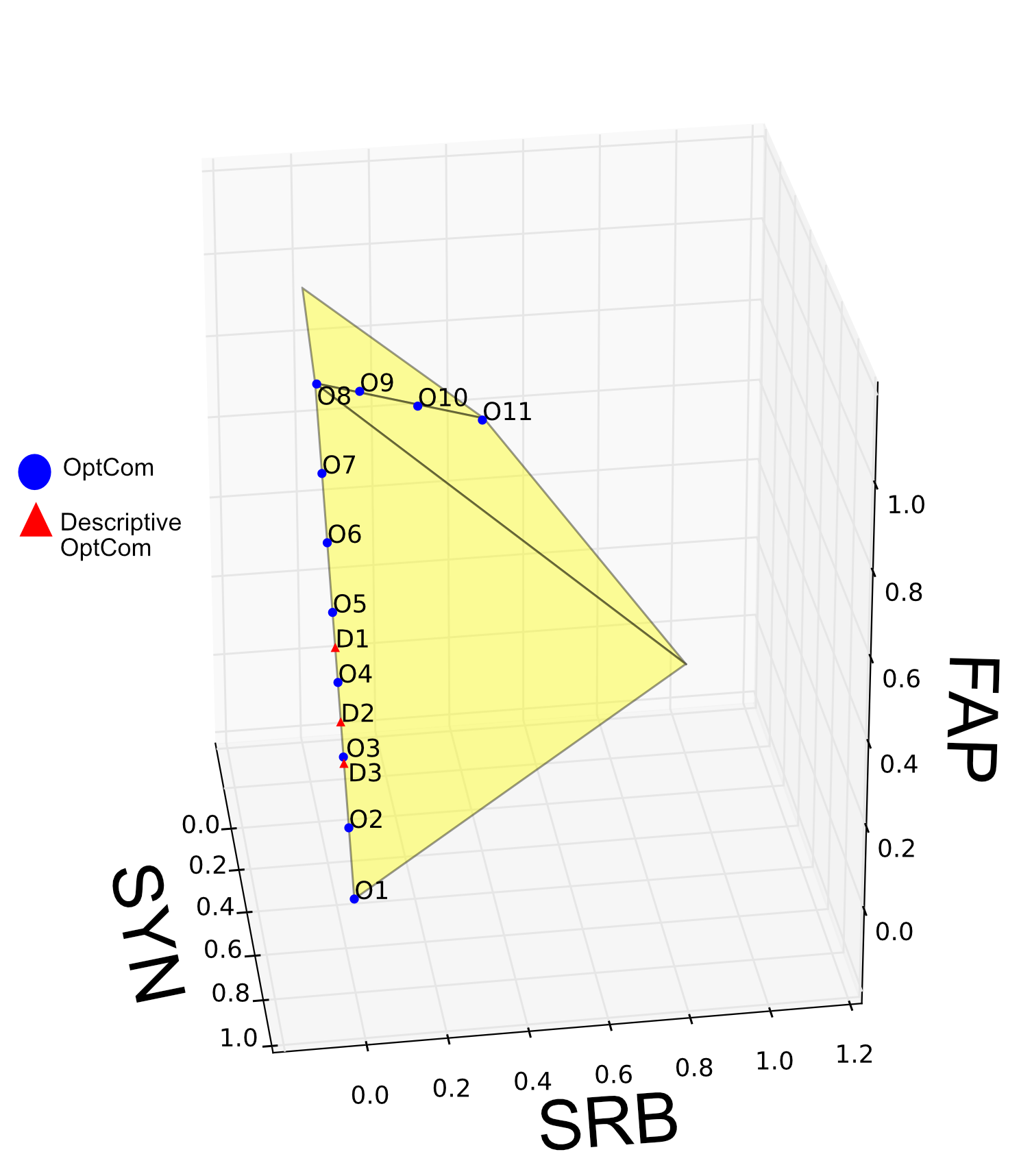

Supplement: S6 Fig — (PNG) [file pone.0171744.s009.png]
